# Cell-attached single-channel recordings in intact prefrontal cortex pyramidal neurons reveal compartmentalized D1/D5 receptor modulation of the persistent sodium current

**DOI:** 10.3389/fncir.2015.00004

**Published:** 2015-02-12

**Authors:** Natalia Gorelova, Jeremy K. Seamans

**Affiliations:** Department of Psychiatry and Brain Research Centre, University of British ColumbiaVancouver, BC, Canada

**Keywords:** prefrontal cortex, Na^+^ channels, single channel recordings, persistent Na^+^ current, dopamine, D1/D5 receptors

## Abstract

The persistent Na^+^ current (*I*_Nap_) is believed to be an important target of dopamine modulation in prefrontal cortex (PFC) neurons. While past studies have tested the effects of dopamine on *I*_Nap_, the results have been contradictory largely because of difficulties in measuring *I*_Nap_ using somatic whole-cell recordings. To circumvent these confounds we used the cell-attached patch-clamp technique to record single Na^+^ channels from the soma, proximal dendrite (PD) or proximal axon (PA) of intact prefrontal layer V pyramidal neurons. Under baseline conditions, numerous well resolved Na^+^ channel openings were recorded that exhibited an extrapolated reversal potential of 73 mV, a slope conductance of 14–19 pS and were blocked by tetrodotoxin (TTX). While similar in most respects, the propensity to exhibit prolonged bursts lasting >40 ms was many fold greater in the axon than the soma or dendrite. Bath application of the D1/D5 receptor agonist SKF81297 shifted the ensemble current activation curve leftward and increased the number of late events recorded from the PD but not the soma or PA. However, the greatest effect was on prolonged bursting where the D1/D5 receptor agonist increased their occurrence 3 fold in the PD and nearly 7 fold in the soma, but not at all in the PA. As a result, D1/D5 receptor activation equalized the probability of prolonged burst occurrence across the proximal axosomatodendritic region. Therefore, D1/D5 receptor modulation appears to be targeted mainly to Na^+^ channels in the PD/soma and not the PA. By circumventing the pitfalls of previous attempts to study the D1/D5 receptor modulation of *I*_Nap_, we demonstrate conclusively that D1/D5 receptor activation can increase the *I*_Nap_ generated proximally, however questions still remain as to how D1/D5 receptor modulates Na^+^ currents in the more distal initial segment where most of the *I*_Nap_ is normally generated.

## Introduction

Dopamine modulates a number of cognitive functions mediated by the prefrontal cortex (PFC) while dysregulation of the mesocortical dopamine system is thought to occur in psychiatric conditions. One current that plays an important role in shaping PFC activity is the persistent Na^+^ current (*I*_Nap_). *I*_Nap_ is similar to the fast transient Na^+^ current but tends to activate at a lower voltage and inactivates more slowly (French and Gage, 1985; Patlak and Ortiz, 1985; French et al., 1990; Alzheimer et al., 1993; Taylor, 1993; Astman et al., 2006). *I*_Nap_ strongly regulates intrinsic excitability, membrane oscillations (White et al., 1998; Hu et al., 2009), synaptic amplification (Stuart and Sakmann, 1995) and persistent activity (Durstewitz et al., 2000) while computational modeling has suggested that *I*_Nap_ neuromodulation can profoundly affect overall network activity (Durstewitz et al., 2000; Durstewitz and Seamans, 2008).

A number of studies have reported that dopamine modulates *I*_Nap_ in PFC neurons but the issue has been quite contentious. The inconsistencies may stem largely from the limitations of the techniques commonly used to study *I*_Nap_. In the initial papers, sharp intracellular pipettes were used (Geijo-Barrientos and Pastore, 1995; Yang and Seamans, 1996) which create a considerable shunt around the electrode and extremely poor voltage control. A subsequent study used whole-cell patch-clamp recordings (Gorelova and Yang, 2000) which provided better, but still imperfect voltage control given the expansive dendritic arbor of deep layer PFC pyramidal neurons. One way to circumvent this problem, employed by Maurice et al. (2001) was to use dissociated cells where the neurites were enzymatically and mechanically severed. However, given the diameter of the axon relative to the soma, if the axon is >10 um in length, it’s voltage is still difficult to control from a somatic electrode (White et al., 1995) while a second drawback is that key intracellular cascades could be disrupted or lost in the dissociation procedure which could be potentially serious given the dramatic differences in *I*_Nap_ in the presence vs. absence of various intracellular molecules (Ma et al., 1994, 1997; Fleidervish et al., 2008). A final problem is that each Na^+^ channel subtype tends to be distributed nonuniformly throughout the axonal-somato-dendritic region (Raman and Bean, 1997; Smith et al., 1998; Caldwell et al., 2000; Goldin, 2001; Rush et al., 2005; Osorio et al., 2010). Since all studies of dopamine modulation of *I*_Nap_ to date have recorded exclusively from the soma, the issue of compartmentalized modulation has not been experimentally addressed.

The only solution to this myriad of potential artifacts and complications is to employ a technique where one can record in different cellular compartments with perfect voltage control while leaving intracellular signaling cascades untouched. This is possible with the cell-attached recording configuration. In the present study we performed cell-attached recordings from the soma, proximal apical dendrite (PD) and the proximal axon (PA) of deep layer PFC neurons. Using this approach we tested the effects of a D1/D5 receptor agonist on multiple aspects of Na^+^ channel gating in hopes of gaining new insights into this controversial issue.

## Methods

### Slice preparation

The use and care of animals as well as protocol for slice preparation from anesthetized rats were approved by University of British Columbia Animal Care Committee.

Slices containing the medial prefrontal cortex (mPFC) were prepared from brains of 16–26 day old Sprague-Dawley rats. Animals were anesthetized with Isoflurane and killed by decapitation. The brain was quickly removed and placed in ice-cold oxygenated (CO_2_ 95%, O_2_ 5%) cutting solution containing (in mM): 120 NaCl, 20 NaHCO_3_, 10 HEPES, 3 NaOH, 2.5 KCl, 9 MgCl_2_, 0.5 CaCl_2_, 25 D-glucose, 0.4 L-ascorbic acid. Coronal slices containing mPFC were cut on a vibratome at 300 µm. Dissected slices were kept at room temperature in a holding chamber in continuously oxygenated artificial cerebrospinal solution (ACSF) containing (in mM) 125 NaCl, 25 NaHCO_3_, 2.5 KCl, 2 CaCl_2_, 1 MgCl_2_, 1.25 NaH_2_PO_4_, 25 D-glucose, 0.4 L-ascorbic acid and 0.01 CNQX. The same composition ASCF was used for recording. After >1 h incubation, slices were transferred to a recording chamber and perfused with continuously oxygenated ACSF at a rate of 1–1.5 ml/min. Recordings were made at room temperature.

### Pharmacological agents

Stock solutions of CNQX, AP5 (Ascent Scientific, Princeton USA), TTX (Alomone labs, Israel) and SKF81297 (Sigma) were prepared in water, aliquoted and stored frozen at −30°C. Each drug was thawed and diluted to an appropriate concentration immediately before application.

### Single channel recordings

**Figure 1:**
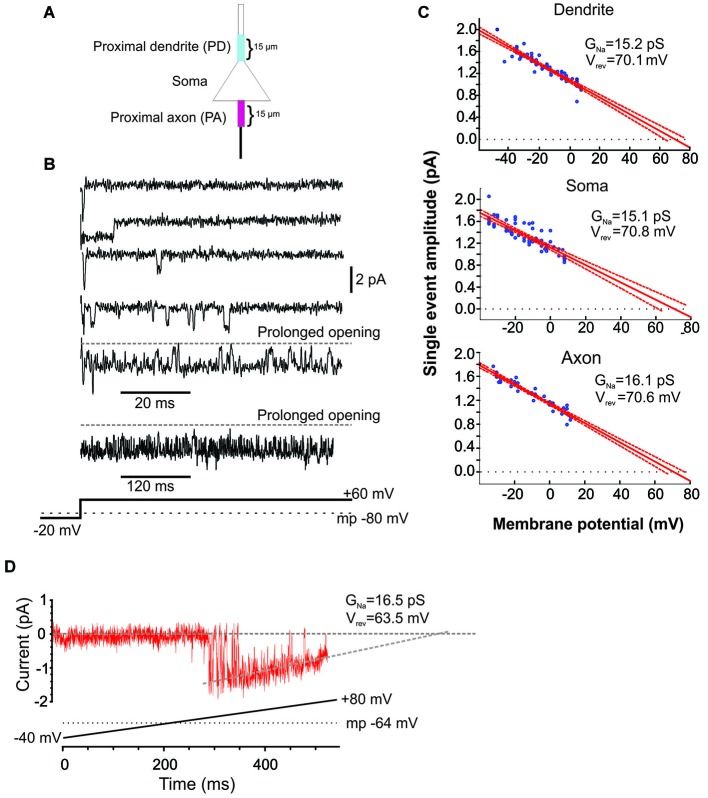
**Characteristics of Na^+^ channel gating in mPFC neurons. (A)** Schematic of the recording arrangement. Cell-attached patch-clamp recordings were made from the soma, the proximal dendrite (PD) or the proximal axon (PA), within 15 um of the soma. **(B)** Example traces from a cell-attached recording of Na^+^ channel openings in the PD evoked by a +60 mV voltage step from a holding potential −20 mV below the presumed resting membrane potential of −80 mV (illustrated in the bottom schematic). Openings varied widely in duration. In some cases prolonged bursts were recorded that lasted hundreds of ms. **(C)** Group plot of the slope conductances derived from all recordings from the PD (top), soma (middle) or PA (bottom). The x-axis gives the transmembrane potential to which the patch was stepped (starting from a holding potential −40 mV hyperpolarized from rest) and the y-axis gives the average amplitude of all single openings >2 ms in duration evoked by the step. Each blue dot is data from a single patch and the red line is the regression fit (with 95% confidence intervals) to the dots. The extrapolated slope conductance and reversal potentials are provided in the insets. **(D)** An example PD patch recording in which prolonged burst events were sufficiently frequent so as to allow for an investigation of the current throughout a voltage ramp. Channel openings began at an approximate transmembrane potential of −45 mV and decreased in amplitude as the driving force collapsed. The extrapolated slope conductance and reversal potential are given in the inset. The voltage ramp protocol is given in the bottom schematic and involved holding the patch −40 mV below rest and sweeping the voltage to 80 mV above rest. The resting transmembrane potential, obtained after break in, is given by the dotted gray line.

Layer V pyramidal cells were visualized in brain slices using infrared differential interference contrast optics (Axioskop Zeiss). Recordings were made from cell bodies, proximal apical dendrites (PD, 5–10 µm from soma) and proximal axons (PA, axon initial segment, 3–15 µm from soma) (Figure 1A). Pipettes were brought next to the neuron and very weak positive pressure was used to clean the surface before seal formation. Single channel recordings were made in cell-attached configuration. Patch pipettes were made from thick wall borosilicate glass capillaries with an outer diameter of 1.5 mm. The internal surface of the glass capillaries was treated with Sigmacote and allowed to dry at room temperature at least 3 days before being used for manufacturing patch pipettes. This treatment significantly reduced capacitance and improved the quality of the seal, which approached values >40 GΩ. To reduce the number of single channels in a patch we used pipettes with resistances of 15–25 MΩ when filled with patch solution. The pipette solution for recording Na^+^ channels contained the following (in mM): 130 NaCl, 3 KCl, 2 CaCl_2_, 2 MgCl_2_, 0.1 CdCl_2_, 0.02 CNQX, 0.05 AP5, 10 D-glucose, 5 tetraethylammonium chloride, 1 4-AP and 10 HEPES with a pH of 7.3. The pipette solution for recording delayed rectifier K^+^ channels contained the following (in mM); 150 KCl, 10 HEPES, 2 CaCl_2_, 2 MgCl_2_, 10 D-glucose with a pH of 7.4.

Command voltage protocols were generated and single-channel currents were acquired using an Axopatch 200 B amplifier with a Digidata 1320A analog-to-digital interface (Axon Instruments, CA). Capacitive transients were minimized using built-in circuits of the amplifier. Data were low-pass filtered at 2 or 5 kHz and digitized at 50 kHz. The root mean square (RMS) noise was usually between 0.125 and 0.25 pA. Patches were held 20–40 mV more negative than the resting membrane potential and stepped to potentials 20–80 mV more positive than the resting membrane potential.

### Data analysis

Data were analyzed using Clampfit 9.0 and 10.4 (pClamp package, Axon Instruments). Residual capacitance transits were nullified by off-line subtraction. For detection of single channels, state transitions with a minimum duration threshold of 0.05 ms were used. A list of idealized channel events was created and used for further analysis. For deriving single channel conductances, the amplitudes of well resolved square shape unitary events were chosen and the amplitudes of 15–25 unitary events measured at a given membrane potential were plotted against membrane potential for each patch. To calculate a slope conductance and extrapolated reversal potential, a linear regression analysis was performed in Statistica. For calculating the conductance of channels entering the prolonged bursting mode, we used the following depolarizing voltage ramp: from a holding potential 40 mV more negative than resting membrane potential, the voltage was slowly increased to 80 mV more positive than resting membrane potential at a rate of 0.2 mV/ms. Traces without channel openings were averaged and this average trace was used for leak subtraction.

Ensemble-average traces were constructed by averaging 60 individual sweeps. The peak current at each potential was then converted to a conductance assuming a Na^+^ reversal potential of +60 mV. Least square fits to the Boltzmann function:

y=A/{1+exp[(Vm−V1/2)/k]}+C

were made in Clampfit for each individual patch as well as for groups of patches.

A repeated measures ANOVA was used for statistical analysis of the voltage dependance of brief late Na^+^ channel openings in the PD, soma and PA. A Student’s *t*-test was used to determine the significance of the effect of the D1/D5 receptor agonist on ensemble currents. For statistical analysis of the effect of the D1/D5 receptor agonist on the brief late Na^+^ channel openings and their gating, Student’s *t*-tests with Holm-Bonferroni correction for multiple comparisons were performed. The values in the text and figures are presented as mean ± SEM. The degrees of freedom are presented as the subscripts to *F* and *t*.

## Results

### Baseline characteristics of unitary and ensemble Na^+^ channel currents in mPFC neurons

The present study includes 22 cell-attached recordings from the soma, 34 from the PD and 13 from the proximal axon (PA; Figure 1). Even though all recordings were performed in cell-attached mode, inward currents were shown as downward for consistency.

The ability to analyze and compare cell-attached recordings from different sites or under different conditions requires a reasonably accurate knowledge of the transmembrane potential. This can be difficult for cell-attached recordings. Following each recording, we applied suction to attain whole-cell mode and quickly recorded the membrane potential. The average resting voltage at break in was −72.1 ± 0.7 mV, *n* = 23. The membrane potential at break-in was used as a correction in all of the analyses described below.

Since the pipette solution contained blockers of K^+^ (TEA, CsCl), Ca^2+^ currents (CdCl), AMPA (CNQX) and NMDA (AP5) currents, the remaining inwardly going single channel openings were assumed to be Na^+^ currents. Accordingly, when the selective blocker of Na^+^ channels, TTX (1 uM), was included in patch solution, no inward single channel openings were observed (*n* = 11, not shown). Examples of Na^+^ channel gating in a PD cell-attached recording is shown in Figure 1B. From a presumed holding potential of −100 mV, 80 mV voltage steps produced early channel openings as well as multiple late channel openings. Openings included single brief openings, short bursts of brief openings as well as prolonged burst openings. The amplitudes of the brief yet fully resolved late (>20 ms after voltage step initiation) single openings were quantified across a family of voltage steps and plotted against the membrane potential (Figure 1C). The slope of regression line gave us the conductance of unitary openings and the extrapolated reversal potential. The reversal potentials calculated for 11 patches were between +68.8 and +79.1 mV, with an average +73.1 ± 0.96 mV, *n* = 11. This is very close to the calculated Nernst equilibration potential for Na^+^ current at 25°C which would be +66.9 to 76 mV with an external Na^+^ concentration (i.e., the patch solution) of 135 mM and assuming an internal Na^+^ concentration of 7–10 mM.

Using the same approach we also calculated the slope conductances of the Na^+^ channels recorded from the three regions. The average conductance of late single events recorded from PD recordings was 15.2 ± 3.8 pS, *n* = 8, from somatic recordings was 15.1 ± 3.2 pS, *n* = 8 and from PA recordings was 16.1 ± 4.8 pS, *n* = 7. While combining many patches in this manner was useful in that it produced robust overall estimates, it could occlude subtle differences in the individual slope conductances present in a given patch. While most patches had Na^+^ channels with conductances of ~16 pS, there were a few patches from the axon and dendrite (but not soma) that exhibited a slope conductance of ~19 pS. These conductance values are very consistent with past studies of *I*_Nap_ in cultured cortical pyramidal neurons (Magistretti et al., 1999a,b; Magistretti and Alonso, 2006).

In addition to the brief late openings, the channels sometimes exhibited prolonged burst openings that could last several hundreds of milliseconds (Figure 1B). To attain a measure of the conductance of channels displaying sustained burst openings, we exploited the prolonged nature of these bursts and recorded channel openings during depolarizing voltage ramps from a transmembrane potential of −120 mV to 0 mV. An example of one of these prolonged burst openings recorded from a dendritic patch during a depolarizing ramp is shown in Figure 1D. Regression analysis yielded a slope conductance of 16.5 pS for the patch shown in Figure 1D and an average value of 16.7 ± 2.96 pS for 5 additional PD patches. This conductance value was very consistent with what was obtained from single events shown in Figure 1C. Therefore, the present results suggest that *I*_Nap_ in layer V PFC neurons can be produced by a population of ~16 pS Na^+^ channels that enter a distinct prolonged gating mode, consistent with past studies in neurons from other cortical regions (Alzheimer et al., 1993).

All patches contained multiple channels as manifest by the appearance of overlapping multiple openings at the beginning of the depolarizing steps. To combine or compare data obtained from different patches we estimated the number of channels in each patch using peak current variance methods. Assuming that all Na^+^ channels within a patch are independent and have uniform conductance and open probability, the number of channels (*N*) and the peak open probability (*P_o_*) can be derived as follows (Kimitsuki et al., 1990; Astman et al., 2006):

$$N=I_{\text{peak}}/iP_{\text{o}}$$

$$P_{o}=1-\sigma_{\text{peak}}^{2}/iI_{\text{peak}}$$

where *I*_peak_ is the average Na^+^ current value at the peak, *σ*^2^ is the peak Na^+^ current variance and i is the unitary single channel current amplitude.

To estimate the number of channels in each patch we measured the amplitude of the peak current during a 60 mV depolarizing step as well as the later unitary single channel currents that occurred from 20 ms to the end of the step. Across the entire data set, there was an average of 5.9 ± 0.8, *n* = 16 channels/patch in somatic patches, 7.6 ± 1.5, *n* = 18 channels/patch in PD patches and 9.5 ± 1.8, *n* = 13 channels/patch in PA patches. For the analysis of late openings, we normalized the number of openings and open probability obtained for each patch based on the estimated number of channels in the patch.

The late channel openings were counted starting 20 ms after the beginning of the depolarizing step. The average number of late openings per channel per sweep was calculated by dividing the number of all late openings by the number of channels in the patch and by the number of depolarizing sweeps. Open probability of late openings was calculated as a ratio of the total open time during depolarizing steps relative to the total time of the depolarization and then divided by the estimated number of channels in the patch. To obtain the voltage dependance of late openings, patches were held 20 mV more negative than the resting membrane potential and stepped to potentials 20–80 mV more positive than the resting membrane potential in 5 mV intervals (corrected based on the resting membrane potential at break-in).

**Figure 2:**
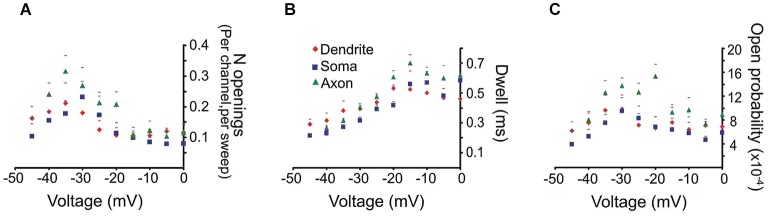
**Quantitative analysis of the late Na^+^ channel openings**. The late channel openings were counted starting 20 ms after the beginning of the depolarizing step. Each panel provides the average group data from PD patches (red diamonds), somatic patches (blue squares) or PA patches (green triangles). The SEM is given by the corresponding colored lines. **(A)** The number of late Na^+^ channel openings (per channel, per sweep) (*N*) **(B)** dwell time or **(C)** open probability (*P*_o_) of late Na^+^ channel openings for each region as a function of transmembrane voltage.

To derive mean values of the number of openings, dwell time and *P*_o_ we combined data from different patches in 5 mV bins. The mean number of openings, dwell time and *P*_o_ for events recorded from the three regions are shown in Figure 2. We included in the analysis all late single openings or late openings that appeared as a part of brief bursts. Bursts with durations longer than 40 ms were excluded from this analysis but will be dealt with below. For all regions the largest number of openings was observed at an estimated transmembrane voltage of −30 to −40 mV. The mean number of openings was not significantly different for the three areas (*F*_(2,8)_ = 0.98, *p* = 0.41). The mean dwell time progressively increased with larger step voltages and attained an asymptote at ~−20 mV. Again the three regions did not differ in terms of mean dwell time (*F*_(2,8)_ = 1.77, *p* = 0.22). Finally the mean *P*_o_ peaked at ~−30 mV and also did not show a difference between the regions after Holm-Bonferroni correction for multiple comparisons (*F*_(2,8)_ = 4.3, *p* = 0.049).

**Figure 3:**
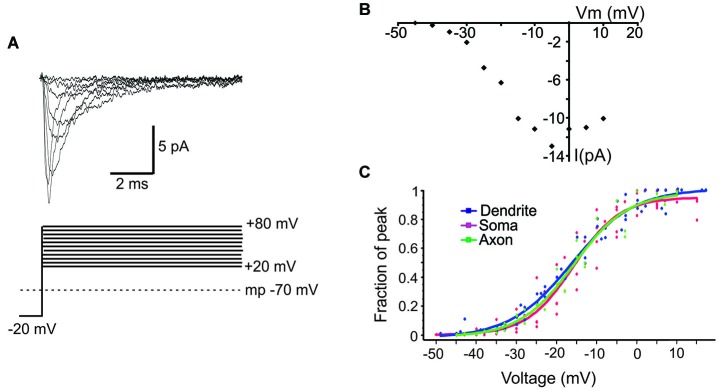
**Properties of ensemble Na^+^ currents. (A)** Representative recordings (top) from a PD patch showing the ensemble Na^+^ currents evoked by various amplitude voltage steps from a holding potential −20 mV hyperpolarized from rest. Each line is an average of >60 individual traces. The transmembrane potential is given by the gray dotted line in the bottom schematic. **(B)** The I-V plot of the patch shown in **(A)**. In this graph the *x*-axis is the transmembrane voltage to which the patch was stepped and the *y*-axis is the peak single channel current. **(C)** Plots of normalized peak conductances as a function of steps to various transmembrane potentials for groups of patches from the PD (blue), soma (purple) and PA (green). Each dot represents the normalized conductance for a single patch. The lines are Boltzmann fits.

Next we characterized the ensemble currents produced by summing over numerous single sweeps (Figure 3A). For these experiments patches were held −40 mV below rest and a series of voltage steps 20–80 mV above rest were delivered. Even for patches with the smallest N, an ensemble current could always be observed by averaging hundreds traces following a voltage step to −20 mV. However, for constructing I-V plots we only used patches containing more than 6 channels. Figure 3B describes the I-V relationship of the ensemble current depicted in Figure 3A. We used two approaches to calculate the average half activation voltage (*V*_mid_) for each region. First, Boltzmann fits to the normalized conductances for each patch were performed and the average *V*_mid_ was then calculated. The resultant *V*_mid_ values were not different between regions: −16.1 ± 1.11 mV, *n* = 6 for the PD vs. −16.4 ± 2.65 mV, *n* = 5 for the soma vs. 16.5 ± mV, *n* = 5 for the PA (*F*_(2,14)_ = 0.04, *p* = 0.96). Second, for each region we combined the normalized conductance values from all single patches into a single plot and then performed the Boltzmann fits (Figure 3C). The obtained values of *V*_mid_ were similar to the first approach and were −16.4 ± 0.65 mV for the PD, −16.2 ± 1.04 mV for the soma and 16.14 ± 0.72 for the PA.

### D1/D5 receptor modulation of unitary and ensemble Na^+^ channel currents in mPFC neurons

**Figure 4:**
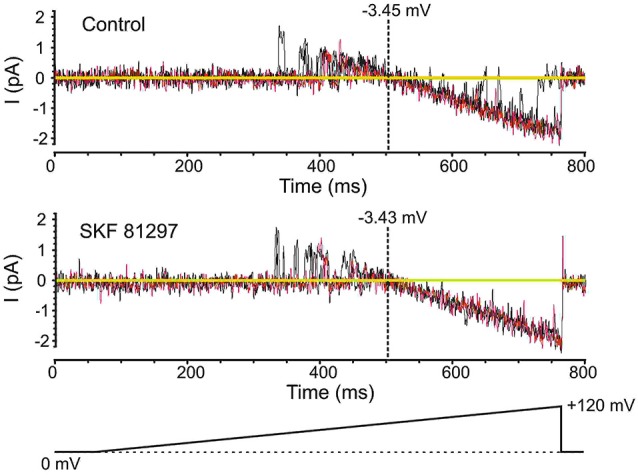
**Testing the effects of SKF81297 on membrane potential based on an analysis of K^+^ channels**. To get a surrogate measure of transmembrane voltage in cell-attached mode, the reversal potential for delayed rectifier K^+^ channel openings was used. For these experiments, the patch solutions were altered by removing K^+^ channel blockers and matching the [K^+^] in the patch pipette to the intracellular concentration, yielding a reversal potential near 0 mV. Voltage ramps started at the resting membrane potential and moved to +120 mV depolarized from rest (bottom schematic). The resting membrane potential for the presented cell was −80 mV. Multiple continuous openings were evoked. These openings started as outward but flipped to inward as the patch was depolarized. The reversal occurred at a transmembrane potential of −3.45 mV (top). Following the bath application of SKF81297 (3–5 µM), the reversal occurred at a transmembrane potential of −3.43 mV (bottom). Black and red lines are single sweeps. Sweeps with channel openings across a wide range of voltages were chosen. The background current was subtracted.

Prior to analyzing the effects of the D1/D5 receptor agonist SKF81297 on Na^+^ channel gating, it was important to determine whether the drug affected the membrane potential, since a change in voltage would alter all voltage-dependent measurements. To test this the K^+^ reversal potential was analyzed under baseline conditions and following the administration of SKF81297 (3–5 µM) in the bath. The delayed rectifier K^+^ current was chosen because it is very prominent in cell-attached recordings from mPFC neurons in the absence of TEA. To measure changes in K^+^ reversal potential, we recorded the delayed rectifier K^+^ channel using a patch solution with a potassium concentration of 150 mM. This was close to the internal potassium concentration, thereby bringing the K^+^ reversal potential in the patch close to 0. We used the following ramping voltage protocol: from the resting membrane potential, the voltage was slowly increased to 120 mV more positive than the resting membrane potential at a rate of 0.2 mV/ms. By delivering such ramping protocols it allowed us directly record the reversal potential of the current with an accuracy of ±0.5 mV. In 5 patches tested, the K^+^ reversal potential changed by less than 1 mV (range −0.8 mV + 0.6 mV) following D1/D5 receptor agonist administration (Figure 4). This indicated that any impact of SKF81297 on membrane potential was negligible and should not contaminate our analysis of its effects on *I*_Nap_.

The effect of the D1/D5 receptor agonist on Na^+^ channel gating was assessed in two ways. Since it was difficult to attain a viable patch with unwavering seal resistance for more than ~15 min, there was usually insufficient opportunity to measure Na^+^ channel gating across a variety of voltage steps under baseline and SKF81297 conditions in the same patch. Therefore, we either tested a single voltage step under baseline conditions and following SKF81297 in a single patch, or we performed a series of voltage steps in one group of patches under control conditions and repeated the same voltage steps in a different group of patches that received SKF81297 immediately upon seal stabilization.

**Figure 5:**
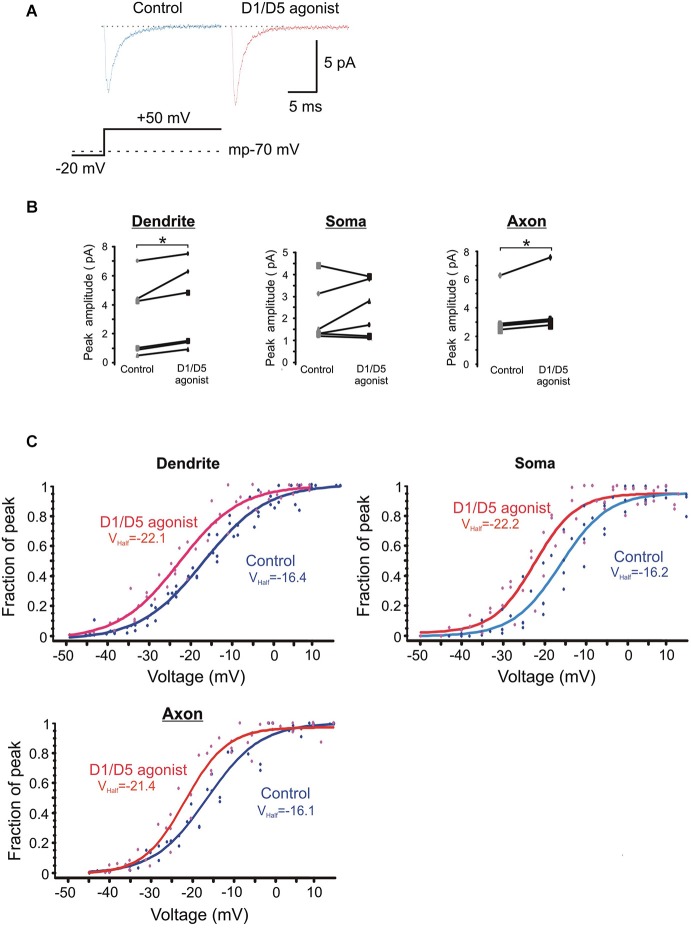
**The effects of the D1/D5 agonist on the ensemble Na^+^ current. (A)** Representative traces showing the ensemble current evoked by the voltage step shown in the bottom schematic under control conditions (left, blue) and following bath application of SKF81297 (3 µM) (right, red). The resting membrane potential for this patch recorded after break-in is given by the dotted line in the bottom schematic. **(B)** Change in the average ensemble Na^+^ current amplitude evoked by a 50 mV voltage step above rest in single patches by SKF81297. Each dot represents the averaged ensemble Na^+^ current amplitude recorded for a single patch. Patches were stepped to a single voltage under control conditions and following bath application of SKF81297 (3 µM). * represents significance at *p* < 0.05. **(C)** Plots of the normalized peak conductances as a function of steps to various transmembrane potentials for PD, somatic and PA patches. Each dot represents the normalized peak conductance for a single patch. Lines represent Boltzmann fits under control conditions (blue) and in SKF81297 (red). Average half activation is given in the insets.

The average ensemble response from a single PD patch under baseline and SKF81297 conditions is shown in Figure 5A for a voltage step to a transmembrane potential of −20 mV. It shows a moderate increase in the ensemble current in response to D1/D5 receptor stimulation. Figure 5B represents group data for the patches from the three regions. The amplitudes of the ensemble currents were increased by SKF81297 in PD patches by 28 ± 8 %, *n* = 6, in somatic patches by 23 ± 17 %, *n* = 6 and in PA patches by 25 ± 7 %, *n* = 5.

The normalized conductances were then plotted as a function of voltage for the group of patches recorded under baseline conditions and a different group of patches recorded in the presence of 3 µM SKF81297. Boltzmann fits revealed that SKF81297 shifted the Na^+^ current activation curve leftward in all three regions (Figure 5C). The same analysis was rerun in a slightly different manner in that the Boltzmann fits were performed first on each patch and then the results were combined. This also showed that SKF81297 had a significant effect on *V*_mid_ in the PD (−16.3 ± 2.7 mV, *n* = 6 in control vs. −22.6 ± 3.6 mV, *n* = 7 in SKF81297, *t*_11_ = 3.79, *p* < 0.01), the soma (−16.1 ± 4.4 mV, *n* = 5 in control vs. −21.4 ± 3.2 mV, *n* = 5 in SKF81297 *t*_8_ = 2.43, *p* < 0.05) and the PA (−16.6 ± 2.5 mV, *n* = 5 in control vs. −21.6 ± 0.75 mV, *n* = 5 in SKF81297, *t*_8_ = 4.44, *p* < 0.01). There were no significant differences in the average maximal current amplitudes between the control group of patches and the patches treated with SKF81297 (PD: 7.3 ± 4.8 pA, *n* = 6 in control vs. 10.3 ± 5.2 pA, *n* = 7 in SKF81297, *t*_11_ = 1.1, *p* = 0.29) (soma: 6.7 ± 1.8 pA, *n* = 5 in control vs. 6.1 ± 1.8 pA, *n* = 5 in SKF81297, *t*_8_ = 0.54, *p* = 0.6) (PA:15.1 ± 8.2 pA, *n* = 5 in control vs. 14.04 ± 2.9 pA, *n* = 5 in SKF81297, *t*_8_ = 0.21, *p* = 0.84). Therefore, based on this analysis of ensemble currents, D1/D5 receptor activation caused a greater Na^+^ current for the same voltage step because it produced a leftward shift in activation, rather than an absolute increase in the peak channel conductance. In these experiments, the average membrane potential at break in was −71.8 ± 0.7 mV, *n* = 18 for all cells in the control group and did not differ significantly from the average membrane potential at break-in for all cells treated with SKF81297 (−71.2 ± 0.4 mV, *n* = 18) (*t*_34_ = −0.8, *p* = 0.43).

**Figure 6:**
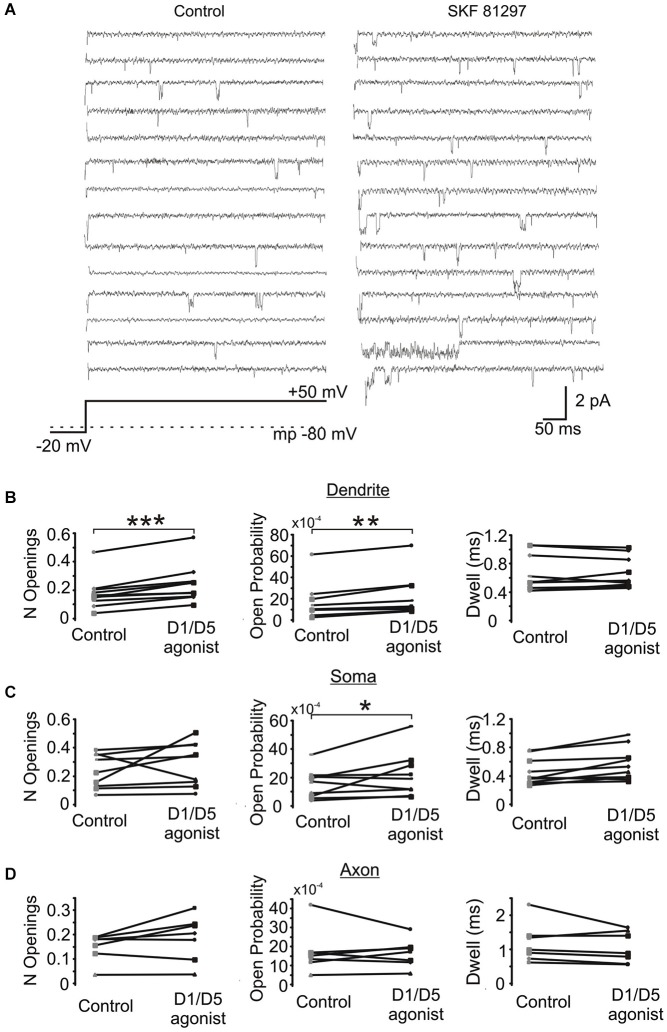
**The effect of a D1/D5 agonist on Na^+^ channel gating. (A)** Traces from a representative PD patch where isolated Na^+^ channel openings were evoked by 70 mV voltage steps from −100 mV to −30 mV (see bottom schematic). The resting potential is given by the dotted line in the bottom schematic. Control traces are shown at the left and traces from the same patch following bath application of SKF81297(3 µM) are given at the right. **(B)** Quantification of the effects of the D1/D5 agonist on the late openings of Na^+^ channels recorded from the PD: the number of late openings (per channel, per 80 ms) (left), the open probability (middle) or the average dwell time (right). The late channel openings were counted starting 20 ms after the beginning of the depolarizing step. Each pair of connected dots is from a single patch. **(C)** Same as **(B)** but for patches recorded from the soma. **(D)** Same as **(B)** but for patches recorded from the PA. ***represents significance at *p* < 0.001, **represents significance at *p* < 0.01 and *represents significance at *p* < 0.05.

Next we investigated the effects of SKF81297 on multiple late single channel openings. In these experiments we utilized 100 ms and 550 ms depolarizing voltage steps. Since we didn’t find any difference in the late Na^+^ channel openings between these two groups, they were pooled. The number of late openings was calculated by dividing the number of all late openings by the number of channels in the patch and by the number of depolarizing sweeps and then scaled to a 80 ms length of sweep. Figure 6A shows example traces from a dendritic patch under baseline conditions and following the activation of D1 receptors by SKF81297. Across the population of patches recorded at voltage steps to transmembrane potentials of −40 to −50 mV, SKF81297 significantly increased the number of openings in the PD (0.17 ± 0.036 in control vs. 0.24 ± 0.042 in SKF81297, *t*_9_ = 5.96, *p* = 0.0001), but not the soma (0.24 ± 0.04 in control vs. 0.29 ± 0.051 in SKF81297, *t*_8_ = 1.15, *p* = 0.14) or PA (0.148 ± 0.021 in control vs. 0.18 ± 0.035 in SKF81297, *t*_6_ = 1.84, *p* = 0.057) (Figure 6B). SKF81297 also significantly increased *P*_o_ in the PD (0.00165 ± 0.00054 in control vs. 0.00217 ± 0.00068 in SKF81297, *t*_9_ = 3.75, *p* < 0.002) the soma (0.00155 ± 0.0003 in control vs. 0.00217 ± 0.00053 in SKF81297, *t*_8_ = 2.41, *p* = 0.045) but not the PA (0.00168 ± 0.00044 in control vs. 0.00160 ± 0.00028 in SKF81297, *t*_6_ = −0.33, *p* = 0.37) (Figures 6B,C). The overall dwell time did not differ under baseline vs. SKF81297 (Figures 6B–D) in the PD (0.663 ± 0.081 ms in control vs. 0.656 ± 0.068 ms in SKF81297, *t*_9_ = 0.31, *p* = 0.38), the soma (0.519 ± 0.058 ms in control vs. 0.621 ± 0.072 ms in SKF81297, *t*_8_ = 2.52, *p* = 0.05) or the PA (0.944 ± 0.158 ms in control vs. 0.982 ± 0.119 ms in SKF81297, *t*_6_ = −1.27, *p* = 0.12). Therefore, D1/D5 receptor stimulation mainly increased the probability that Na^+^ channels open in the PD and to a lesser extent in the soma.

**Figure 7:**
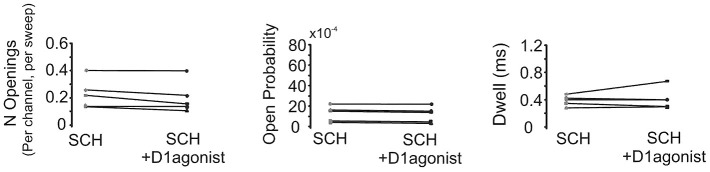
**The D1/D5 receptor antagonist blocks the effect of the D1/D5 agonist on the late Na^+^ channel openings**. Quantification of the effects of the D1/D5 agonist (3 µM SKF81297) in the presence of the D1/D5 receptor antagonist (3 µM SCH23390) on the average number of late openings (per channel, per sweep) (left), the open probability (middle) and the average dwell time (right) of single Na^+^ channels recorded from the PD during a 70 mV depolarizing step. Patches were held 20 mV more negative than resting membrane potential. Each pair of connected dots represents data from the same patch.

In order to confirm that the above effect of SKF81297 on Na^+^ channel gating in the PD was due to D1/D5 receptor activation, we tested if the D1/D5 receptor antagonist SCH23390 could block the effect of SKF81297 by applying SCH23390 (3 µM) 10 min before application of SKF81297(3 µM). As can be seen in Figure 7, when the D1/D5 receptor agonist was applied in the presence of a D1/D5 receptor antagonist, no increase in either the number of late channel openings (0.24 ± 0.0049 in SCH23390 control vs. 0.21 ± 0.052 in SKF81297 + SCH23390, *t*_4_ = 2.23, *p* = 0.09), the channel open probability (0.0015 ± 0.0003 in SCH23390 control vs. 0.0014 ± 0.0003 in SKF81297 + SCH23390, *t*_4_ = 4.7, *p* = 0.009) or the dwell time (0.457 ± 0.037 ms in SCH23390 control vs. 0.486 ± 0.073 ms in SKF81297 + SCH23390, *t*_4_ = 0.66, *p* = 0.55) was observed.

Next we compared the effects of the D1/D5 receptor agonist on the number of single openings vs. short bursts (prolonged bursts will be dealt with separately). For these analyses, short bursts were defined as multiple events occurring within an interval <2 ms and with a total duration of less than 40 ms. SKF81297 had marginal but non-significant effects on the number of isolated single events in the PD (0.082 ± 0.015 in control vs. 0.097 ± 0.021 in SKF81297, *t*_9_ = 1.44, *p* = 0.09), the soma (0.089 ± 0.016 in control vs. 0.094 ± 0.019 in SKF81297, *t*_8_ = 0.48,*p* = 0.32) and the PA (0.049 ± 0.033 in control vs. 0.062 ± 0.014 in SKF81297, *t*_6_ = 2.02, *p* = 0.05). In contrast, the D1/D5 receptor agonist affected various burst metrics as shown in Table 1. Specifically, D1/D5 receptor stimulation significantly increased the total number of short bursts but only in the PD (0.034 ± 0.0093 in control vs. 0.053 ± 0.014 in SKF81297, *t*_9_ = 6.07, *p* = 0.00009, Table 1). It also increased the total number of events that occurred within all the recorded bursts, but again only in the PD (0.097 ± 0.028 in control vs. 0.146 ± 0.033 in SKF81297, *t*_9_ = 5.43 *p* = 0.0002, Table 1). In contrast, the D1/D5 receptor agonist did not affect the average number of events/burst (2.51 ± 0.48 in control vs. 2.78 ± 0.75 in SKF81297, *t*_9_ = 0.95, *p* = 0.09, Table 1). Thus the most likely explanation for these results was that SKF81297 caused an enhanced propensity of the Na^+^ channel to open in bursts.

**Table 1:** **Analysis of various burst properties affected by the D1/D5 receptor agonist**.

	PD		Soma		PA		All	
	%	*p*-value	%	*p*-value	%	*p*-value	%	*p*-value
Single events	19	0.09	12	0.32	22.3	0.05	17.5	0.02
Bursts	97	0.00009	19.6	0.31	7	0.22	43.8	0.02
Event count/burst	10.8	0.09	14	0.003	7.6	0.048	11	0.002
Events occurring in bursts	98.7	0.0002	66	0.056	16.4	0.14	65.2	0.0006

*For each region, the left column is the percent change from baseline and the right column is the p-value as determined by paired sample t-tests. The degrees of freedom are 9 for the dendrite, 8 for the soma and 6 for the axon. Alpha levels were determined by Holm-Bonferroni correction*.

**Figure 8:**
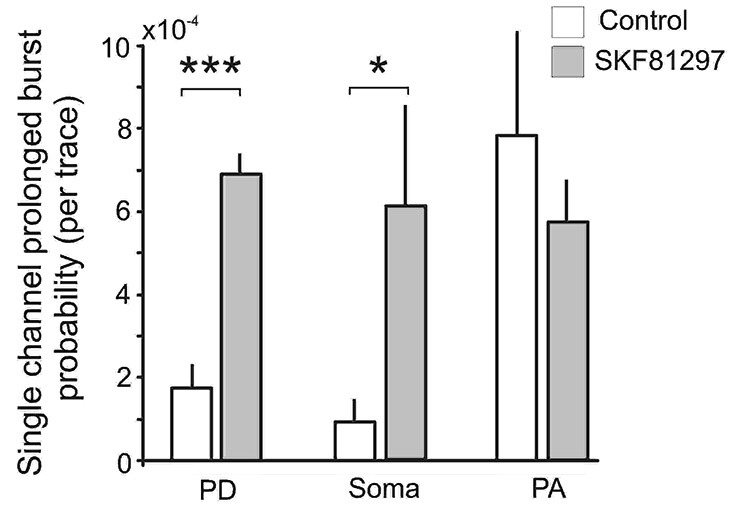
**The effects of the D1/D5 receptor agonist on prolonged burst openings**. Prolonged burst openings were openings of Na^+^ channels that lasted >40 ms. Such bursts were recorded when the patch was stepped from −20 mV below rest to 50 mV above rest under control conditions (open bars). Although the frequencies were highest in the PA under baseline conditions, SKF81297 (3 µM) increased the probability that they would occur in the soma and PD but not in the PA. ***represents significance at *p* < 0.001, *represents significance at *p* < 0.01.

Finally we analyzed the effect of SKF81297 on prolonged bursts. First we analyzed the probability of channel entering the prolonged burst mode in patches subjected to 50 mV depolarizing steps in control and during D1/D5 agonist application. For each region we calculated the number of prolonged openings for all patches and divided this number by the total number of traces multiplied by the number of channels in each patch. The probability of prolonged burst were higher during D1/D5 agonist application compared to the control in the PD (0.000282 in control vs. 0.000783 in SKF81297) and the soma (0.000212 in control vs. 0.001575 in SKF81297) but not the PA (0.000562 in control vs. 0.000631 in SKF81297). The low probability of prolonged bursts prevented us from performing statistical comparisons on these data. To overcome this, we calculated the probability of prolonged openings in control patches and in a separate group of patches that were exposed to the D1/D5 receptor agonist. Each patch was subjected to a series of steps to several membrane potentials, totaling ~1000 traces for each patch. The probability of prolonged bursts was calculated for each patch. Even though prolonged bursts were many fold more prevalent in the PA than the PD or soma under baseline conditions, SKF81297 increased the mean probability of their occurrence only in the PD (*t*_12_ = 6.42, *p* = 0.0003) and the soma (*t*_10_ = 2.36, *p* = 0.01) but not the PA (*t*_10_ = −0.59, *p* = 0.48) (Figure 8). In fact, the D1/D5 receptor agonist brought the prevalence of prolonged bursts in the PD and soma to the level of the PA under baseline conditions (Figure 8) and therefore selectively boosted the relative impact of *I*_Nap_ in these regions. This tendency to promote prolonged bursting was the most significant effect of the D1/D5 receptor agonist on *I*_Nap_ overall yet is very consistent with the conclusion above, that the drug also increased the propensity of Na^+^ channels to open in shorter bursts.

Given these findings, it is of interest to consider how the recorded channels might contribute to the whole cell *I*_Nap_ under control conditions and following SKF81297. The total *I*_Nap_ current for each region was estimated based on Na^+^ channel kinetics for steps to −20 mV using the following equation:

$$I_{\text{Nap}}=N*\left(P_{\text{oB}}+P_{\text{oL}}\right)*i,$$

where *N* is the total number of channels, *P*_oB_ is the open probability for brief openings, *P*_oL_ is the open probability for prolonged burst openings and *i* is the unitary current amplitude. Although we estimated the number of channels in each patch from the actual recordings and used this value as a means to make conclusions about the single channel properties, our experimentally derived values for *N* were not used in the calculation of the whole-cell *I*_Nap_, since we were not exactly certain of the area of our patches and because the cytoskeletal properties of each region may differently affect the number of channels/patch (Kole et al., 2008). Rather, the determination of N was based on the published properties of cortical pyramidal neurons. Assuming the soma of a layer V cortical pyramidal cell is ~20 µm wide and 25 µm long, it possesses a total surface area of 1099 µm^2^ (for a cone). A PD ~5 µm in diameter and cylindrical, would have a surface area 314 µm^2^ for a 20 µm length, whereas an axon ~1.2 µm in diameter, would have a surface area of 75 µm^2^ for a 20 µm length. Sodium channel density has been estimated to be 5 per µm^2^ for the soma and PD (Hu et al., 2009). The estimates of sodium channel density in the initial segment vary from three fold to 40–50 fold higher than that of soma depending on methods used (Colbert and Pan, 2002; Kole et al., 2008; Hu et al., 2009; Fleidervish et al., 2010). For our calculations we used a 10 times higher density of sodium channels in the PA compared to the soma, yielding 50 channels per µm^2^. Therefore, we estimate there would be 5495 Na^+^ channels at the soma, 1570 Na^+^ channels in the first 20 µm of the PD and 3750 Na^+^ channels in the first 20 µm of the PA. In our recordings, the average unitary current amplitude at −20 mV across all the patches was 1.55 pA. *P*_oL_ was calculated using the following equation:

$$P_{\text{oL}}=\sum T_{\text{j}}/\sum t_{\text{j}}*n_{\text{j}},$$

where *T*_j_ is the total time of all prolonged burst openings for the patch *j*, *t_j_* is the total duration of all recorded −20 mV steps for the patch *j* and *n_j_* is the number of channels in patch *j*.

The sums for each region were calculated across all patches subjected to 50 mV depolarizing steps in control and during D1/D5 receptor agonist application. This gave *P*_oL_ values of 0.00028 for the PD, 0.00021 for the soma and 0.00042 for the PA. Values of *P*_oB_ were 0.00165, 0.00155 and 0.00168 for the PD, soma and PA respectively.

Based on these values, under control conditions the contribution of brief late openings to the total *I*_Nap_ would be ~4 pA for the PD, 13.2 pA for the soma and 9.8 pA for the PA while the contribution of prolonged burst openings would be ~0.68 pA for the PD, 1.79 pA for the soma and 2.44 pA for the PA. The combined contribution of brief late openings and prolonged burst openings would be expected to produce a total *I*_Nap_ of ~4.68 pA for the PD, 14.99 pA for the soma and 12.2 pA for the PA. The total *I*_Nap_ across the three regions would be ~31.9 pA, a value that is comparable to that obtained previously in acutely dissociated cortical pyramidal cells (see Maurice et al., 2001).

These calculations were repeated but using values obtained from the same patches in the presence of SKF81297. The *P*_oB_ values during D1/D5 receptor agonist application were 0.00217, 0.00217 and 0.0016 for the PD, soma and PA respectively. And the calculated *P*_oL_ values were 0.00078, 0.0015 and 0.00047 for the PD, soma and PA respectively. In this case, the *I*_Nap_ produced by brief late openings would now be ~5.28 pA for the PD, 18.48 pA for the soma and 9.3 pA for the PA, whereas the *I*_Nap_ resulting from prolonged burst openings would be ~1.9 pA for the PD, 12.8 pA for the soma and 2.73 pA for PA. The total *I*_Nap_ in SKF81297 would therefore be ~7.18 pA for the PD, 31.28 pA for the soma and 12 pA for the PA and when combined across the three regions would produce a total *I*_Nap_ of ~50.46 pA. This represents a 60% increase over control. Furthermore, under control conditions prolonged bursts would contribute only ~15% of total *I*_Nap_, whereas following SKF81297, the contribution of prolonged bursts would increase to 35%.

## Discussion

The present study investigated the effects of the D1/D5 receptor agonist SKF81297 on single Na^+^ channel gating recorded from the PD, soma and PA of deep layer mPFC neurons in acute brain slices. We found that SKF81297 shifted the activation of the early transient channel openings to more negative potentials in all three regions, while increasing the *P*_o_ of late openings and increasing prolonged burst probability mainly in the PD and to lesser extent in the soma. And as was estimated above, these effects would lead to an increase in the whole-cell *I*_Nap_.

*I*_Nap_ was first demonstrated in neocortical neurons by Stafstrom et al. (1982, 1985). It was initially thought that a prolonged Na^+^ current could be produced by a window current attributable to the overlap between steady-state activation and inactivation (Attwell et al., 1979). Subsequently, *I*_Nap_ has been commonly interpreted to result from brief forays of the fast Na^+^ channel into a persistent or “noninactivating” gating mode during as little as 1% or less of all depolarizations (French and Gage, 1985; Patlak and Ortiz, 1985; French et al., 1990; Alzheimer et al., 1993; Taylor, 1993; Astman et al., 2006). It was proposed that in cortical layer V pyramidal cells, *I*_Nap_ was generated primarily by Na^+^ channels in the axon (Astman et al., 2006) and was attributed to the presence of Nav 1.6 channels (Caldwell et al., 2000; Hu et al., 2009) which enter the noninactivating gating mode more frequently and produce a significantly larger *I*_Nap_ than Nav1.1–1.2 channels localized in the soma and dendrites (Raman and Bean, 1997; Smith et al., 1998; Goldin, 2001; Rush et al., 2005). However, data obtained from Nav 1.6 knock-out mice revealed that although a large proportion of *I*_Nap_ in layer V PFC cells is attributable to Na^+^ channels containing the Nav 1.6 subunit, Na^+^ channels with Nav1.1−1.2 subunits also contribute to *I*_Nap_ (Maurice et al., 2001).

In the present study, the early transient current recorded in the PA displayed the same half activation as the early transient current recorded from soma and PD. Yet Na^+^ channels recorded from PA displayed significantly larger open probabilities than for the PD and soma. Specifically, under control conditions the average probability of prolonged bursts in the PD and soma was 10 times lower than that of the late single or short burst openings. In contrast, in the PA the average probability of prolonged bursts was comparable to that of the late single or short burst openings. This implies that the prolonged bursts make a far greater contribution to the total *I*_Na+_ in the PA. It also suggests that while our recordings were in close proximity to each other, the regions were still functionally segregated in terms of their compliment of Na^+^ channels.

In the studies of Na^+^ channel gating in cultured entrorhinal layer II neurons, the average conductance of persistent burst openings was higher than that of early openings responsible for the transient Na^+^ current (~20 vs. ~15 pS) (Magistretti et al., 1999a,b; Magistretti and Alonso, 2006). While we did detect subgroups of Na^+^ channels with different conductance levels, we found that channels with a conductance of ~16 pS could produce persistent openings. Magistretti et al. (2003) showed that single Na^+^ channels can exhibit at least three “bursting states” of different mean durations but that each Na^+^ channel preferentially operates predominately in a specific gating mode for protracted periods (Magistretti et al., 1999b; Magistretti and Alonso, 2006). These observations raise the perennial question of whether *I*_Nap_ is mediated by differential gating in a common pool of Na^+^ channels or whether distinct Na^+^ channels are responsible for *I*_Nat_ and *I*_Nap_. Magistretti et al. (1999b) argued for the possibility of something in between, as a subgroup of transient Na^+^ channels may undergo some form of modulation to enter prolonged persistent gating modes. Supporting this contention, Szulczyk et al. (2012) recently showed that activation of D1/D5 dopamine receptors increased the availability of the fast Na^+^ current without affecting current amplitude through a cAMP/PCA mechanism in mPFC neurons recorded in cell-attached mode. The present data also support the predictions of Magistretti et al. (1999b). On one hand, the single channel openings themselves were little changed as the single channel amplitude and dwell times in control and SKF81297 conditions were not significantly different. In spite of this, D1/D5 receptor activation significantly increased the number of openings as well as the propensity of the Na^+^ channels to open in short and especially prolonged bursts in the PD and soma. In fact, prolonged burst probability increased three fold in the PD and nearly seven fold in the soma, which effectively brought the probabilities to the levels observed under baseline conditions in the PA. Thus D1/D5 receptor stimulation created a more uniform *I*_Nap_ in mPFC neurons by equalizing basal differences in burst propensity across the soma, axon and dendrite.

Our estimates of the contribution of late openings of Na^+^ channels in the PD, soma and PA to the whole cell *I*_Nap_ show that activation of D1/D5 receptors can lead to a significant increase in the whole cell *I*_Nap_. Although useful as a means to help contextualize the significance of the single channel data, there are some caveats to these estimates that should be borne in mind. First, our estimates of *I*_Nap_ from the PA are not a reliable indicator of the total *I*_Nap_ produced in the initial segment. As shown by Astman et al. (2006), most of the *I*_Nap_ in cortical pyramidal neurons is generated in the distal portion of the initial segment, well beyond where we recorded. In the proximal region of the axon, Nav 1.2 is dominant, rather than Nav 1.6 (Hu et al., 2009) that exits more distally. On the other hand, our estimates of *I*_Nap_ from the soma and PD do not fall prey to this issue since the density of Na^+^ channels does not tend to increase as one moves away from the soma into the dendrites. Therefore, if the peak whole cell *I*_Nap_ recorded from the soma is ~300 pA (Astman et al., 2006), and we estimate that the three proximal compartments collectively generate a ~30 pA *I*_Nap_, then the distal initial segment of the axon must generate the remaining 90% of the whole cell *I*_Nap_. This conclusion is well in line with that of Astman et al. (2006). Hence, in order to attain a comprehensive understanding of dopamine modulation of Na^+^ currents in mPFC neurons, similar cell-attached recordings from Na^+^ channels in the distal axonal initial segment are still required. A second important point is that the “whole-cell” *I*_Nap_ may not always be the key variable of interest as *I*_Nap_ generated in unique compartments might independently contribute to different aspects of signal processing. While the distal Nav 1.6 channels were proposed to be the main spike triggers, Nav 1.2 channels may primarily aid in spike back propagation from the axon to soma (Hu et al., 2009). Dendritic Na^+^ channels might have a completely different function. For example, in an intact brain, overall membrane conductance is expected to be greater during periods of enhanced network activity, making neurons less electrically compact. This will result in a greater attenuation of synaptic potentials approaching the soma and axon along the apical dendrite. This may be one situation where D1/D5 receptor modulation plays a particularly important role, given the dramatic increase in the propensity of dendritic Na^+^ channels to burst following SKF8127.

### The effects of dopamine on *I*_Nap_ in the context of past whole-cell patch-clamp studies

While there is a growing consensus that dopamine acting via D1/D5 receptors increases the excitability of deep layer mPFC neurons, the present data shed some light on the sharp disagreement about whether this change in excitability is related to a change in *I*_Nap_. Initially, Geijo-Barrientos and Pastore (1995) used sharp intracellular recordings in the absence of blockers of other ionic currents to show that dopamine reduced a persistent inward current with properties consistent with *I*_Nap_. Because other ion channels were not blocked, it was difficult to attribute the change directly to *I*_Nap_ modulation however. Soon after Yang and Seamans (1996) used similar recording techniques but found that D1/D5 receptor agonists increased the TTX sensitive Na^+^ plateau potential. A problem with this study was that since sharp somatic electrodes were used, it was impossible to control the voltage of the axo-somato-dendritic region adequately, and although various ion channel blockers were used, the nature of the modulation could not be precisely ascertained. Subsequently, Gorelova and Yang (2000) employed whole-cell patch-clamp recordings in the presence of blockers of most K^+^ and Ca^2+^ channels. They found that D1/D5 receptor agonists shifted the activation of the whole cell *I*_Nap_ leftward and slowed inactivation. Although much better voltage control could be attained with patch electrodes, it was still impossible to control voltage changes in the tiny axon and dendrites from the somatic electrode. Furthermore, a space clamp error by definition means that there is a difference in the potential from the clamped soma to the more distal neurites and therefore a flow of current. In extended pyramidal neuron under these conditions, that flow of current can resemble *I*_Nap_ (White et al., 1995). Maurice et al. (2001) then attempted to circumvent these issues by performing recordings in acutely dissociated mPFC neurons. While they achieved much better voltage control than in past studies, even a length of axon as short as 10 µm can be difficult to control from a somatic pipette (White et al., 1995). In addition, the reported absence of an effect of D1/D5 receptor agonists on *I*_Nap_ could potentially have been the result of a loss/disruption of critical molecules needed for D1/D5 receptor modulation during the enzymatic/mechanical dissociation procedure. While Maurice et al. (2001) provided clear evidence that the D1-PKA pathway was functionally intact and able to modulate the fast Na^+^ current in the dissociated neurons, the D1/D5 mediated increase in excitability of intact PFC neurons is thought to be mediated via a PKC and not a PKA mechanism (Chen et al., 2007). A PKC dependent increase in *I*_Nap_ was also reported by Astman et al. (1998) who showed that PKC activation via phorbol esters greatly increased *I*_Nap_ in somatosensory cortical neurons.

Finally a more recent attempt to address the issue was made by Rotaru et al. (2007). They employed a different approach as they investigated the D1/D5 receptor modulation of the EPSP amplification that is mediated mainly by *I*_Nap_ (Stuart and Sakmann, 1995). They reported that D1/D5 receptor agonist reduced the amplification of EPSP waveforms and concluded that this was due to a reduction in *I*_Nap_. While these authors showed that other currents, including *I*_h_ could impact EPSP amplification in separate experiments, they did not investigate the effects of a D1/D5 receptor agonist on EPSP amplification in the presence of an *I*_h_ blocker. Since D1/D5 agonists increase *I*_h_ (Rosenkranz and Johnston, 2006), this could potentially explain the apparent reduction in amplification by a D1/D5 agonist. The simultaneous modulation of *I*_h_ and *I*_Nap_ by D1/D5 receptor stimulation may be held within a tight balance and small differences in experimental procedures could conceivably shift the balance and thereby contribute to the differences across past studies.

The present study was designed to circumvent these past issues by using cell-attached recordings and showed that D1/D5 agonists increased *I*_Nap_ mainly by promoting more robust bursting behavior in the PD and soma. While uncontaminated by the same issues that plagued past studies, we did not record from the distal initial segment where most of the *I*_Nap_ is generated. Therefore, while the present data are quite clear in terms of how D1/D5 receptor activations modulates Na^+^ channels proximal to the soma, general statements about how D1/D5 receptors modulate *I*_Nap_ overall and under various realistic conditions, await future investigations.

## Conflict of interest statement

The authors declare that the research was conducted in the absence of any commercial or financial relationships that could be construed as a potential conflict of interest.
